# Motor imagery and electrical stimulation reproduce corticospinal excitability at levels similar to voluntary muscle contraction

**DOI:** 10.1186/1743-0003-11-94

**Published:** 2014-06-05

**Authors:** Fuminari Kaneko, Tatsuya Hayami, Toshiyuki Aoyama, Tomohiro Kizuka

**Affiliations:** 1 Laboratory of SensoryMotor Science and Sports Neuroscience, Second Division of Physical Therapy, Sapporo Medical University, West 17- South 1, Chuo-ku, Sapporo City, Japan; 2 Division of Health Science Education, School of General Education, Shinshu University, Asahi 3-1-1, Matsumoto City, Japan; 3 Department of Physical Therapy, Ibaraki Prefectural University of Health Sciences, 4669-2, Ami, Ami-machi, Inashiki-gun, Ibaraki, Japan; 4 Faculty of Health and Sport Sciences, University of Tsukuba, Tennodai 1-1-1, Tsukuba City, Japan

**Keywords:** Electrical stimulation, Motor imagery, Corticospinal tract, Rehabilitation, Physical therapy

## Abstract

**Background:**

The combination of voluntary effort and functional electrical stimulation (ES) appears to have a greater potential to induce plasticity in the motor cortex than either electrical stimulation or voluntary training alone. However, it is not clear whether the motor commands from the central nervous system, the afferent input from peripheral organs, or both, are indispensable to induce the facilitative effects on cortical excitability. To clarify whether voluntary motor commands enhance corticospinal tract (CoST) excitability during neuromuscular ES, without producing voluntary muscular contraction (VMC), we examined the effect of a combination of motor imagery (MI) and electrical muscular stimulation on CoST excitability using transcranial magnetic stimulation (TMS).

**Methods:**

Eight neurologically healthy male subjects participated in this study. Five conditions (resting, MI, ES, ES + MI [ESMI], and VMC) were established. In the ES condition, a 50-Hz stimulus was applied for 3 to 5 s to the first dorsal interosseous (FDI) while subjects were relaxed. In the MI condition, subjects were instructed to imagine abducting their index finger. In the ESMI condition, ES was applied approximately 1 s after the subject had begun to imagine index finger abduction. In the VMC condition, subjects modulated the force of index finger abduction to match a target level, which was set at the level produced during the ES condition. TMS was applied on the hotspot for FDI, and the amplitude and latency of motor evoked potentials (MEPs) were measured under each condition.

**Results:**

MEP amplitudes during VMC and ESMI were significantly larger than those during other conditions; there was no significant difference in MEP amplitude between these 2 conditions. The latency of MEPs evoked during MI and VMC were significantly shorter than were those evoked during rest and ES.

**Conclusions:**

MEP acutely reinforced in ESMI may indicate that voluntary motor drive markedly contributes to enhance CoST excitability, without actual muscular contraction.

## Background

Many studies have shown that paired associative stimulation with single transcranial magnetic stimulation (TMS) and ES, repetitive transcranial magnetic stimulation (rTMS), or transcranial direct current stimulation can induce either short-term potentiation or a depressive effect in the motor cortex [1-7]. These facilitatory or suppressive effects are believed to accelerate motor recovery in patients with stroke [8,9] because cortical plasticity plays an important role in motor recovery [10,11], as well as motor learning in healthy individuals [12,13]. In clinical situations, however, interventions other than transcranial stimulation are needed to facilitate cortical potentiation because of epilepsy, limited resources, or patient preference. In such cases, we attempt to provide some form of sensory input (e.g., visual input) [14] or ES, which have been used to improve muscular strength for purposes of rehabilitation.

The effect an exercise has on cortical excitability can be augmented by ES [15,16]. Therefore, the combination of voluntary effort and ES appears to have a greater potential to induce plasticity in the motor cortex than either electrical stimulation or voluntary training alone. However, it is not clear whether the motor commands produced in the central nervous system, the afferent input from peripheral organs, or both, are indispensable for cortical facilitation, since motor commands and afferent input are summed in the cerebral network during voluntary effort. We hypothesize that motor commands (e.g., those evoked during motor imagery [MI]) independently increase the effect of ES on cortical excitability without afferent input, since the increment of corticomotor excitability during MI is widely accepted [17-20]. We propose that the ES potentially affects reflex gain at the spinal level, and via the higher areas of the central nervous system.

In the current study, we used neuromuscular ES combined with MI to examine the acute effects of a combination of ES and voluntary motor commands on cortical excitability. We aimed to test the hypothesis that a combination of MI and ES could reproduce corticospinal excitation at levels similar to those occurring during voluntary muscular contraction (VMC).

## Methods

### Subjects

**Figure 1:**
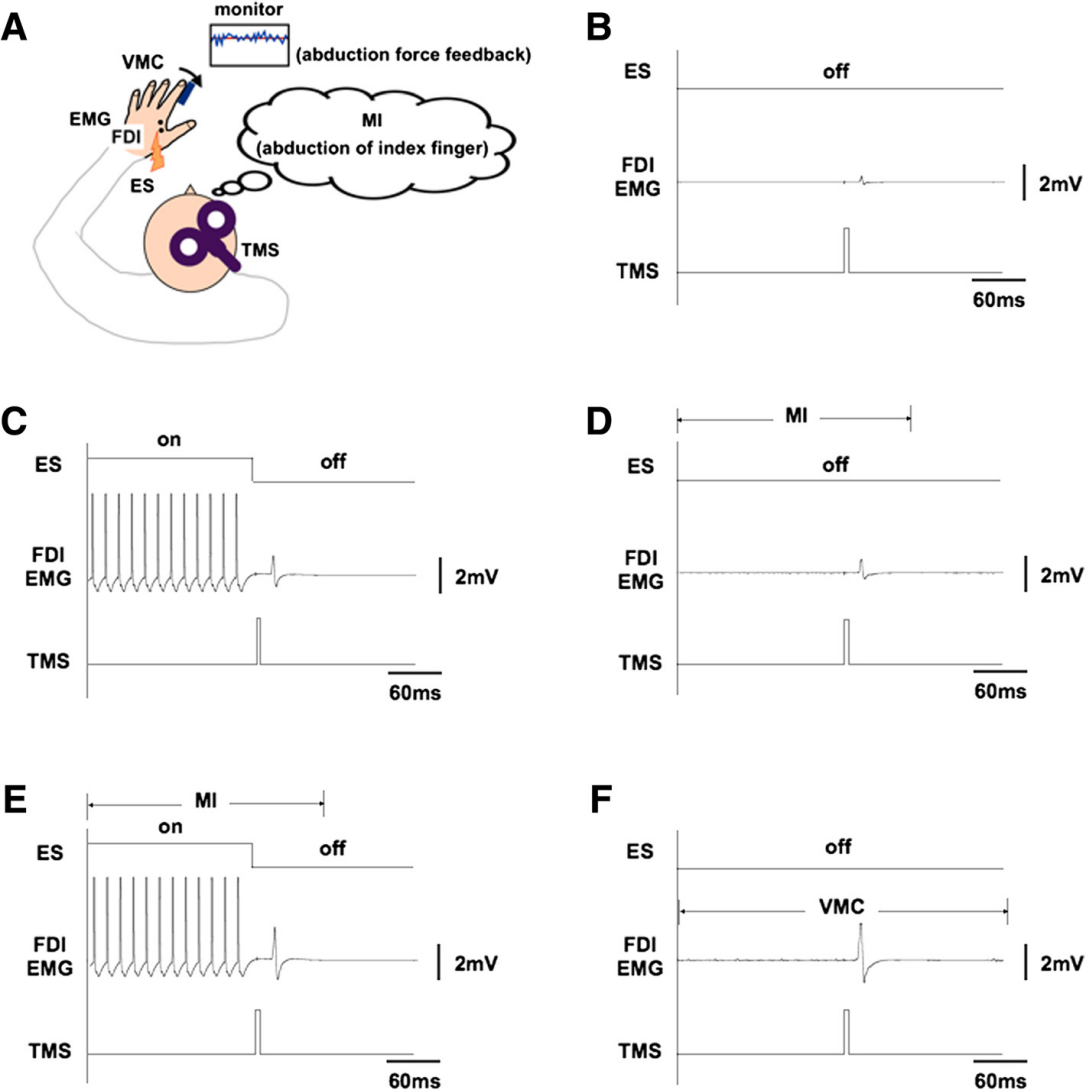
**The experimental setup.** Schematic diagram of the experimental setup **(A)**. TMS was applied during **(B)** rest, **(C)** electrical stimulation (ES), **(D)** motor imagery, **(E)** ES + MI (ESMI), and **(F)** voluntary muscle contraction (VMC). The spike train, which is shown on the left side of the raw first dorsal interosseous (FDI) EMG, was an artifact of ES (C, E); only the last 12 stimuli are shown.

Eight neurologically healthy male subjects (mean age, 23.3 ± 1.8 years; range, 21–26 years) participated in this study. Each subject provided his informed consent prior to participating in the experiment, which was approved by the ethics committee of the National Institute of Advanced Industrial Science and Technology (AIST). This experiment was carried out in the period in which the first author was working in AIST. The experimental setup is shown schematically in Figure 1-A. Subjects were seated comfortably in a chair with their forearm naturally pronated. The left index finger was fixed in the middle position of the full range of abduction, which was the point that subjects felt that they were in the most appropriate position to produce abduction torque.

### Electromyography (EMG)

Surface EMG activity was recorded from the left first dorsal interosseous (FDI) using pairs of Ag-AgCl disc electrodes (5-mm diameter) placed on the center of the muscle belly with a 10-mm interelectrode distance. Prior to placing the electrodes, the skin was cleaned with alcohol and abraded with an abrasive skin-prepping gel. EMG signals were amplified (Neuropack MEB2200, Nihon Kohden Co. Ltd., Tokyo, Japan) (×1000) and filtered (5.3–1000 Hz). The isometric maximum voluntary contraction (MVC) force was first measured for index finger abduction using a force transducer (LMR-S-SA2, Kyowa Electronic Instruments Co., LTD., Tokyo, Japan), which also measured the abduction torque during voluntarily index finger abduction. The EMG signals and index finger abduction torque were digitized and recorded at 20 kHz using an A/D converter (Power 1401, Cambridge Electronic Design, Cambridge, UK). The triggers for ES and TMS were generated by the Power 1401 and temporally synchronized with the EMG and torque signals.

### TMS

TMS (Magstim 200^2^, The Magstim Company Limited, Whitland, UK) was delivered to the right (contralateral) motor cortex, with anteromedial current flow in the motor cortex (perpendicular to the central sulcus), to induce motor evoked potentials (MEPs) recorded from the left FDI. Stimulation was delivered through a figure-eight coil (9-cm diameter loops). Resting motor thresholds were defined as the lowest currents at which MEPs were evoked with a peak-to-peak amplitude greater than 50 μV [21,22]. During testing, the TMS stimulus intensity capable of inducing MEPs with average peak-to-peak amplitudes of 0.5–1 mV was applied during the resting condition. Ten MEPs were recorded under each of 5 conditions (Rest, ES, MI, ES with MI [ESMI], and VMC). To allow us to normalize MEP amplitudes between subjects, we measured the gain of the neuromuscular pathway through to the FDI in each subject after TMS testing. A 1-ms rectangular electrical stimulus was applied to the ulnar nerve at the dorsal point of the medial epicondyle, and the resulting supramaximal M-waves were recorded from the FDI using paired electrodes.

### Experimental conditions

Five conditions were established, including the resting condition (Figure 1-B). In the ES and ESMI conditions (Figure 1-C), 1-ms rectangular electrical pulses were applied at 50 Hz while subjects were relaxed. The duration of the electrical stimulus trains was precisely varied from 2 to 4 s under the control of the Labview software package (National Instruments Corporation, Austin, TX, USA). The duration was an integral number of seconds, the exact value being randomly set in each trial. The stimulation electrodes were placed on the skin next to the recording electrodes and oriented parallel to the muscle fibers. The intensity of ES was set at a level that induced a small percentage of the FDI MVC, measured before the experiment, 5.8 ± 1.0 mA) without eliciting pain. The stimulus intensity of ES was determined at the beginning of the experiment by increasing the ES intensity until it reached the maximum level without causing discomfort or pain. While determining the ES intensity, index finger abduction torque was measured to determine the target torque for the VMC and ESMI conditions, so that the force produced from the FDI contraction was similar in ES, ESMI, and VMC conditions. TMS was applied 20 ms after the last ES pulse ended to prevent stimulation artifacts from contaminating the MEPs in ES and ESMI. In the MI condition, subjects imagined abducting the left index finger with maximal effort (Figure 1-D). TMS was applied one second after beginning of MI while subjects imagined performing finger movements and perceiving the kinesthetic feeling of muscular contraction [18]. The background FDI EMG activity was examined by at least 2 investigators to identify voluntary muscle activation. The EMG activity was viewed on 2 computer monitors. The EMG signal was displayed on a scale of 500 μV/division (10 divisions = full scale) on one monitor and on a scale of almost 100 μV/division (maximum scale) on the other monitor [14,23]. A trial was rejected if a small level of muscle activation was observed during the testing. Furthermore, each trial was examined off-line, and trials containing EMG signals with amplitudes that exceeded 100 μV were excluded from the data analysis. The EMG data was rectified off-line and smoothed with a 1-ms moving average to allow comparisons among the experimental conditions. In the ESMI condition (Figure 1-E), ES was applied approximately 1 s after the subject had begun to imagine index finger abduction, so that MI was performed for at least 3 s. The subject was instructed that it can be finished to imagine the movement after TMS was applied in a trial during MI and ESMI. Before applying this condition, the ES intensity had been finely modulated to adjust the induced force level to the target level if the force level produced was different from the target level. The abduction torque was examined by 2 experimenters to eliminate trials in which the torque deviated from the target level. In the VMC condition (Figure 1-F), subjects modulated the force of their index finger abduction to match a target force level, which was presented on a monitor positioned in front of the subject. The target force level was the level produced during the ES condition. On average, the target force level was set at 1.56 ± 0.63% of the MVC of FDI.

### Data analysis

MEP amplitude and latency were measured during each condition. The MEP amplitude was normalized (divided by the supramaximal M-wave [%Mmax] amplitude) for each subject. MEP latency (ms) was defined as the duration between TMS delivery and the onset of an MEP. One-way repeated measures analysis of variance (ANOVA) were used to test the effect of “condition” (Rest, MI, and VMC) on background EMG activity, MEP amplitude, and MEP latency. Post hoc comparisons were made using Tukey’s HSD; the threshold for statistical significance was set at p < 0.05.

## Results

**Figure 2:**
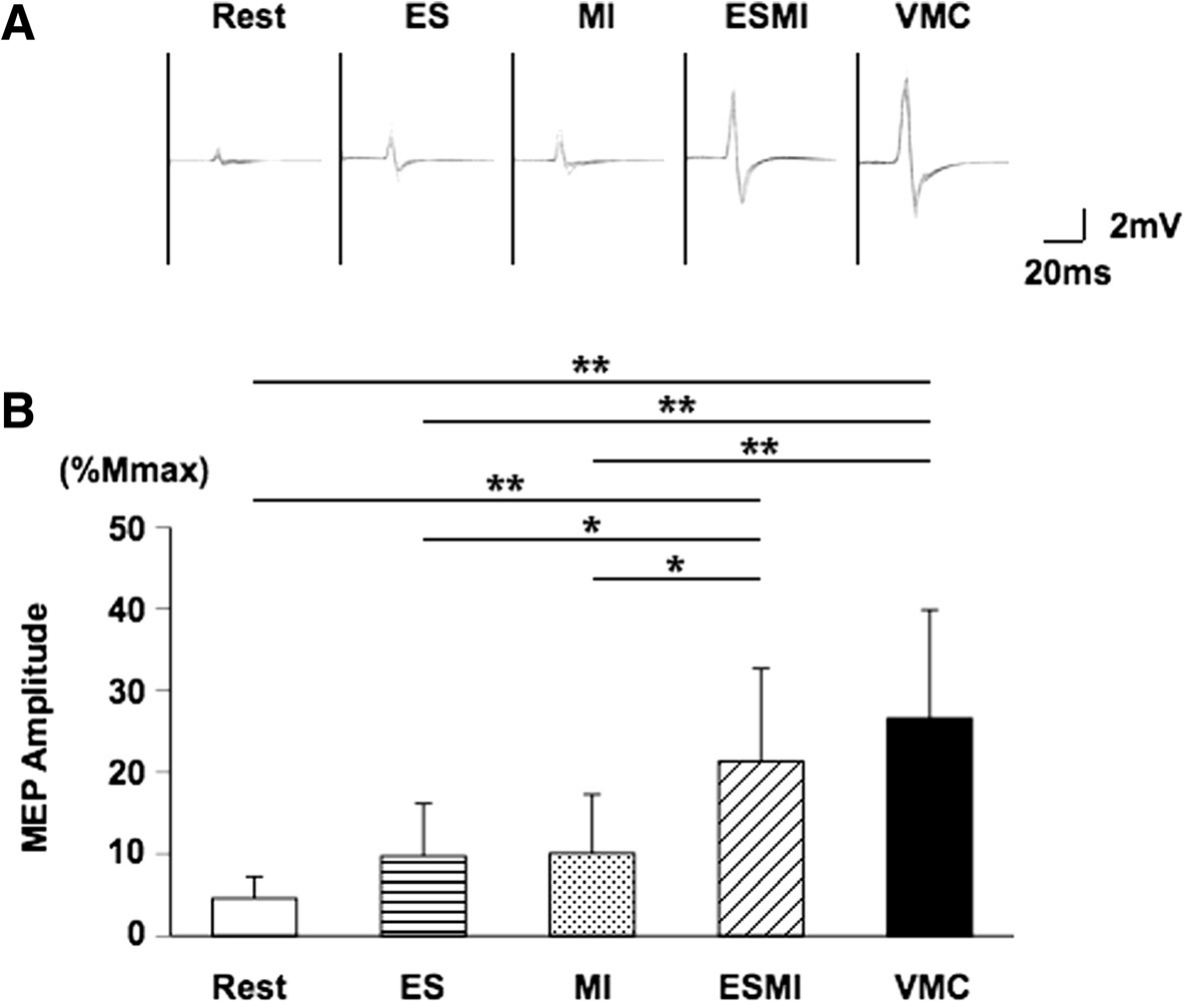
**Motor evoked potential amplitudes.** MEP amplitude recorded from FDI during the resting, ES, MI, ESMI, and VMC conditions. **(A)** MEPs obtained from a single subject in each condition. **(B)** Mean (±S.D.) MEP amplitude recorded in each condition. *p < 0.05, *p < 0.01.

**Figure 3:**
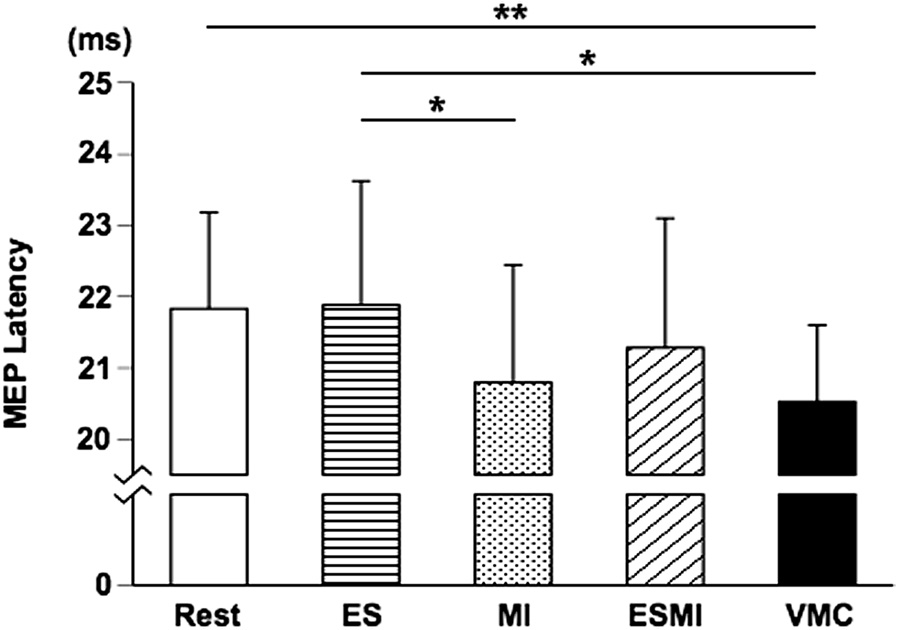
**Mean MEP latencies.** Mean (±S.D.) latency of MEP recorded from FDI in each of the 5 conditions. *p < 0.05, **p < 0.01.

Background EMG activity was compared among the resting, MI, and VMC conditions. The main effect of condition was statistically significant (rest: 0.014 ± 0.002 mV, MI: 0.020 ± 0.004 mV, VMC: 0.043 ± 0.013 mV, F = 32.413, p < 0.0001). Tukey’s HSD post hoc test indicated that EMG activity was greater during VMC than during rest and MI (p < 0.01). There was no significant difference between background EMG at rest and MI.Figure 2-A presents superimposed MEPs recorded during the ES, MI, ESMI, and VMC conditions. The main factor of condition was significant between the 5 different conditions (F = 13.459, p < 0.0001), and Tukey’s HSD post hoc test revealed that MEP amplitudes were significantly increased during both the ESMI and VMC conditions, as compared with the resting, ES, and MI conditions. There was no significant difference in MEP amplitudes between the ESMI and VMC conditions, or among the resting condition and the ES or MI conditions (Figure 2-B). The average MEP amplitudes recorded during ES and MI conditions were larger than those recorded during the resting condition. However, there were no significant differences among the resting, ES, and MI conditions (Figure 2-B). Figure 3 shows the MEP latency for all 5 conditions. The latency in the VMC condition was shorter than those in the resting or ES conditions (Figure 3). The latency was significantly shorter during the MI condition than during the ES condition.

## Discussion

In the present study, TMS revealed a significant facilitation of corticospinal excitability during the ESMI and VMC conditions. More importantly, corticospinal tract excitability was acutely increased in the ESMI condition. MEP amplitude reached a level similar to that measured during brief voluntary muscle contraction (VMC in the present study), even though voluntary muscle contractions were absent during the ESMI condition. This suggests that ESMI intervention may potentiate corticospinal tract excitability. However, the mechanisms through which MEPs were affected in the ESMI and VMC conditions remain unclear, as this factor was not addressed in the current study. This augmented corticospinal tract excitability may be clinically effective for inducing voluntary movement in patients with motor dysfunction, such as patients who have suffered a stroke. Many studies have reported that potentiation after noninvasive cerebral stimuli was effective in motor learning, even if the intended subject had suffered a stroke [8,9,12,13]. rTMS, which can enhance corticospinal excitability [24,25], is not necessarily easy to handle because of its risk of causing epilepsy [21]. If the acutely enhanced excitability shown in the present study were associated with cortical plasticity, then this intervention might be an effective rehabilitation strategy.

The effect on the excitability change during ESMI was clearly different from that seen during either individual ES or MI. The mechanism underlying the reinforcement of corticospinal tract excitability during ESMI may be associated with brain activity that occurs with MI, naturalistic afferent input from the muscle spindles, or spindle and/or cutaneous afferent spiking directly evoked with ES. MI, however, is not a purely psychological intervention. In agreement with several previous studies, the present study confirmed that MI could elicit neural activation in the cortical motor areas [26-28]. We speculate that neural activation was evoked in the cortical motor areas during the MI condition because the average MEP amplitude was greater during the MI condition than during the resting condition, even if the difference between those 2 conditions was not statistically significant. The reinforcement of corticospinal tract excitability does not contradict previous reports [18-20]. In our previous study, we demonstrated the stretch reflex gain increased significantly during MI, without concurrent changes in H-reflex amplitude [23]. We interpreted that finding to suggest that gamma motoneurons received input from the motor cortex during MI, which subsequently increased the stretch reflex gain. If this interpretation is correct and intrafusal muscle fibers contracted as a result of gamma motoneuron activity during MI, the afferent input from the muscle spindle during ES was potentially enhanced when MI was performed simultaneously, than during ES performed without MI. Therefore, in the ESMI condition, the ES-evoked muscle contractions may augment the stretch reflex gain. Furthermore, it is well established that the cortical activation that occurs during MI is similar to that occurs during VMC [29]. Thus, cortical activation is induced by MI, and an enhanced stretch reflex within the spinal circuitry, since the spinal motoneuron pool excitability was enhanced accompanying with the enhanced stretch reflex, may augment corticospinal tract excitability at a level similar to that observed during VMC.

In contrast, ES evokes compound muscular action potentials, and because muscle fibers generally shorten when they contract, the muscle spindles are unloaded during ES. From this perspective, Ia afferent input evoked during ES would differ from that evoked during VMCs. From a different perspective, index finger abduction produced torque during these isometric contractions, and the index finger angle remained unchanged. However, the changes in muscle fiber length in situ that accompanies muscular contraction should occur in synchrony with each electrical pulse; each shortening–lengthening cycle of the muscle must therefore include the muscle extension phase during the relaxation period after contraction. In fact, McKeon and Burke showed that muscle spindles discharge immediately after a single motor unit twitch evoked with an electrical stimulus, even if the muscle was fully relaxed during the stimulation [30]. Consequently, it is difficult to eliminate the possibility that afferent input from the muscle spindle enhanced MEP amplitude during ES. Furthermore, it is possible that the afferent input evoked with ES might be affected at the spinal or cortical level. The mechanisms underlying our findings should be investigated in detail in future studies.

Previous studies have shown that changes in corticospinal excitability were induced with ES applied during VMC [16,31]; however, whether activity in the motor areas produced during VMC, regardless of the state of muscular contraction is important, has not yet been clarified. A significant point in this study is the suggestion that cerebral activity during MI plays an important role, in addition to the effect of ES, in changing corticospinal excitability, regardless of the VMC in the periphery.

The MEP latency was shorter during MI than during ES. A potential reason for this difference in MEP latency is that MEP transmission from the motor cortex was modulated at different anatomical locations between the 2 conditions. Previous studies have shown that neural activity increases in the motor areas during MI [17-20,26-28], suggesting that the MEP increase evoked during MI is likely due to cerebral activation. Furthermore, a shortened latency was noted during VMC; however, the latency in ESMI was unclear, even though MEP amplitude obviously increased. The difference in MEP latency may be due to differences in the anatomical level at which motoneuron excitability was modulated.

One limitation of this study was that voluntary muscle activity during the ESMI condition was not analyzed, whereas the absence of muscular activity was recognized during the MI condition. Furthermore, the absence of EMG was confirmed, at least during the periods when only MI was executed before ES had begun in the ESMI condition. The mechanism of corticospinal excitability reinforcement during ESMI was not investigated in detail. However, it was shown that the combination of intention and ES increased corticospinal excitability, and this may prove to be clinically useful. For example, when functional neuromuscular electrical stimulation would be applied to a patient with stroke to facilitate the cortcicospinal excitability in the involved side, applying it with motor imagery or intension of a movement should be recommended.

## Conclusions

Although the exact mechanisms remain unclear, we successfully reproduced augmented corticospinal excitability during ESMI at a level similar to that observed during VMC. Future studies are necessary to reveal whether sustaining this condition can cause short-term potentiation at the cortical level and is clinically useful in motor rehabilitation.

## Abbreviations

CoST: Corticospinal tract; Rest: Resting condition; MI: Motor imagery; ES: Electrical stimulation; ESMI: Electrical stimulation with MI; VMC: Voluntary muscular contraction; FDI: First dorsal interosseous muscle; MEP: Motor evoked potential; rTMS: Repetitive tanscranial magnetic stimulation; EMG: Electromyography; MVC: Maximum voluntary contraction; %Mmax: MEP amplitude was normalized by the supramaximal M-wave.

## Competing interests

This manuscript has not been published or presented elsewhere in part or in entirety, and is not under consideration by another journal. All study participants provided informed consent, and the study design was approved by the appropriate ethics review boards. All the authors have approved the manuscript and agree with submission to your esteemed journal. There are no conflicts of interest to declare.

## Authors’ contributions

FK and TK conceived the study, participated in the study design and coordination, and helped to draft the manuscript. TA and TH participated in the design of the study and performed the statistical analysis. All authors read and approved the final manuscript.
